# Structure and Mechanical Properties of Shipbuilding Steel Obtained by Direct Laser Deposition and Cold Rolling

**DOI:** 10.3390/ma14237393

**Published:** 2021-12-02

**Authors:** Ruslan Mendagaliyev, Oleg Zotov, Rudolf Korsmik, Grigoriy Zadykyan, Nadezhda Lebedeva, Olga Klimova-Korsmik

**Affiliations:** 1 World-Class Research Center “Advanced Digital Technologies”, St. Petersburg State Marine Technical University, 190121 St. Petersburg, Russia; r.korsmik@ltc.ru (R.K.); lebedevamtm@gmail.com (N.L.); o.klimova@ltc.ru (O.K.-K.); 2 Institute of Metallurgy, Mechanical Engineering and Transport, Peter the Great St. Petersburg Polytechnic University, 195251 St. Petersburg, Russia; zog-58@mail.ru (O.Z.); gzadykyan@mail.ru (G.Z.); 3 CRISM “Prometey” NRC “KI”, 191015 St. Petersburg, Russia

**Keywords:** direct laser deposition (DLD), direct metal deposition (DMD), additive manufacturing (AM), bainitic steels, cold-resistant steels, cold rolling, microstructure, heat treatment (HT), mechanical characteristics

## Abstract

The study of the formation of microstructural features of low-alloy bainite-martensitic steel 09CrNi2MoCu are of particular interest in additive technologies. In this paper, we present a study of cold-rolled samples after direct laser deposition (DLD). We investigated deposited samples after cold plastic deformation with different degrees of deformation compression (50, 60 and 70%) of samples from steel 09CrNi2MoCu. The microstructure and mechanical properties of samples in the initial state and after heat treatment (HT) were analyzed and compared with the samples obtained after cold rolling. The effect on static tensile strength and impact toughness at −40 °C in the initial state and after cold rolling was investigated. The mechanical properties and characteristics of fracture in different directions were determined. Optimal modes and the degree of cold rolling deformation compression required to obtain balanced mechanical properties of samples obtained by additive method were determined. The influence of structural components and martensitic-austenitic phase on the microhardness and mechanical properties of the obtained samples was determined.

## 1. Introduction

With the development of modern production technologies, it has become possible to obtain products using additive technologies [1]. Additive manufacturing technologies are still at the implementation stage. The most common manufacturing methods still use traditional technologies and combined methods [2]. However, additive processes are increasingly being used in the shipbuilding industry, for example, to manufacture products made of cold-resistant steel [3]. Recently, additive manufacturing (AM) has gradually been introduced in the shipbuilding industry for the production of small- to medium-sized spare parts [4] and it has also been used for layer-by-layer construction of larger structural components such as hollow propeller blades [5], propeller brackets, and foremasts. Thus, some shipbuilding departments are now converting steps in the production process to AM technology in order to reduce production costs and increase component productivity.

To date, the most common materials used in additive manufacturing technologies are titanium-based alloys [6], nickel [7], and other steels [8]. Low-carbon and cold-resistant steels are still at the study stage due to the more complex kinetics of structure formation, which directly depends on the ongoing thermal processes. The peculiarities of the direct laser deposition (DLD) process are high temperature gradients, multiple rapid heating and cooling, residual stresses, and structural heterogeneities. The microstructure of materials, such as grain size and morphology, is very sensitive to the influence of temperature fields and directly affects the microhardness and mechanical properties of obtained materials [9].

For low-carbon steels with low content of alloying elements, produced by the DLD method, it is advisable to apply a subsequent treatment in the form of cold or hot plastic deformation. Such treatment contributes to an increase in strength and toughness, while forming a special structure of steel [10,11]. The characteristic of combined high strength and ductility is achieved by low carbon content, optimum alloying, and cold rolling, which results in fine-dispersed or even nanoscale structures [12]. The stability of steel structures under operational loads can be achieved by technological measures during melting, rolling, thermal, and mechanical processing of rolled steel sheets, providing a reduction in the proportion and size of non-metallic inclusions, grain refinement, and obtaining structures with a chain morphology [13].

The mechanical properties of steels depend primarily on the volume fraction, morphology, size, and properties of the constituent phases. It is well known that during the nucleation and growth of ferrite, carbon atoms diffuse from ferrite to austenite, and the carbon content in non-transformed austenite increases with an increasing volume fraction of ferrite [14]. In addition, bainite plays a major role in microstructure, and as a result high values of ultimate strength and relative elongation can be obtained. Two types of bainite (mainly granular and lath bainite) can be observed in the microstructure. The components of bainite include nonequilibrium bainite ferrite, carbides, and M/A phase consisting of austenite and martensite. M/A phase inclusions with plates of bainite ferrite and carbides located along the plate boundaries form lamellar bainite, whereas granular bainite consists of blocky M/A phase inclusions surrounded by bainite ferrite [15]. The presence of both ferrite and bainite components in the microstructure simultaneously makes it possible to achieve high tensile strengths while maintaining high ductility.

There is significant interest in using AM in the shipbuilding industry for creating materials with improved mechanical properties. Today, the possibility of creating hybrid plants for manufacturing parts using combined production methods is being considered. The technology of direct laser deposition followed by cold or hot rolling can significantly increase the economic potential of parts with high strength properties [5].

In this study, we investigate the microstructure of an alloy directly after DLD and subsequent heat treatment (HT). The mechanical properties and characteristics of fracture in different directions are determined. The influence of the microstructure on the mechanical properties of the obtained samples is revealed. In addition, we studied the deposited samples after cold plastic deformation with different degrees of deformation compression, i.e., 50, 60, and 70%. The optimum modes and degree of cold rolling deformation compression were determined for obtaining balanced mechanical properties of samples obtained by an additive method. The influence of structural components on the microhardness and mechanical properties of the obtained samples was determined. Excellent mechanical properties of additive materials with high tensile strength and relative elongation are obtained.

## 2. Materials and Methods

The initial material used was a powder alloy bainite-martensite steel 09CrNi2MoCu (SphereM, Yekaterinburg, Russia). The chemical composition of the steel and the results of the analysis of the chemical composition of the powder are shown in Table 1. The analysis of chemical composition was carried out by X-ray spectral method using a scanning electron microscope “TESCAN VEGA” (Tescan, Brno, Czech Republic) equipped with an energy dispersive spectrometer “INCAX-MAX” (Tescan, Brno, Czech Republic) and by absorption method using a sulfur and carbon analyzer CS-230 from LECO company (LECO Corporation, St. Joseph, MI, USA) in accordance with GOST 12344-88 (RU).

**Figure 1:**
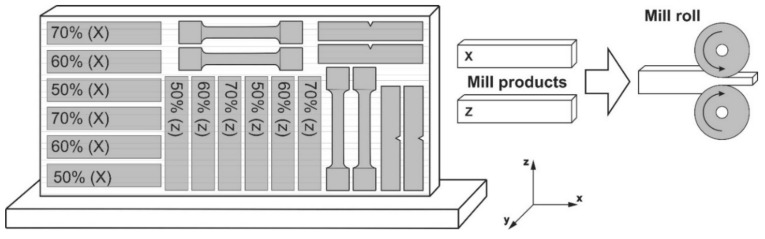
Scheme of cutting and cold rolling of deposited samples.

The samples were deposited at the SPbGMTU experimental facility for DLD. The complex is equipped with the following: industrial robot LRM-200iD_7L (Fanuc, Oshino-mura, Japan), fiber laser LS-3 Yb (IPG Photonics, Oxford, MA, USA), laser sputtering head FLW D30 (IPG Photonics, Oxford, MA, USA), nozzle SO12 (SPbSMTU, St. Petersburg, Russia), and powder feeder Metco Twin 150 (Oerlikon, Freienbach, Switzerland). An argon-filled chamber was used for deposition. The oxygen content in the chamber did not exceed 1000 ppm. The samples were deposited at power P = 2300 W, speed V = 25 mm/s, powder flow rate G = 35 g/min as a transport gas, Δx = 1.67 mm, and Δz = 0.7 mm. A sample of size (L × W × H) 130 × 80 × 15 mm was obtained for the experiment, as shown in Figure 1.

Thermal treatment of deposited samples was performed in a furnace SNOL 30/1300 (Abumega-Group, Utena, Lithuania). The mode of heat treatment was as follows for Mode 1 homogenization: T = 1100 °C exposure for 5 h, hardening at T = 920 °C exposure for 2 h, subsequent cooling in oil, high tempering at T = 650 °C exposure for 6 h, and subsequent cooling in air. Before cold rolling, HT was performed as follows for Mode 2 homogenization: T = 1100 °C with holding time of 1 h and subsequent cooling in the furnace.

Cold-rolled samples were produced on a laboratory mill DUO 220 (Polytechnic University, St. Petersburg, Russia). Cold rolling was performed with 0.5 mm stepwise deformation, up to final crimping of 50%, 60%, and 70%, sample size 60 × 14 × 10 mm. The scheme of cutting and cold rolling is shown in Figure 1.

To reveal the structure, chemical etching of nital (33 mL ethanol + 3 mL HNO_3_) for 5–10 s was performed. For the color etching, Klemm reagent (50 mL of concentrated Na_2_S_2_O_3_ solution + 1 g Na_2_S_2_O_5_) was used for 1–2 min. The deposited samples were examined by optical microscopy on a Leica DMi8 (Leica Microsystems, Wetzlar, Germany) using the “Axalit” program version 3.0 (Axalit, Moscow, Russia) for microstructural analysis. A scanning electron microscope Tescan Mira 3 (Tescan, Brno, Czech Republic) was used for microstructural analysis and fractograms after the mechanical tests.

The contents of different phases in the samples were determined by a method based on etching with Klemm reagent and image analysis using the program “ImageJ”. The volume fraction of bainite, pearlite, and ferrite in the structure was determined according to ASTM E1245 in automated mode.

The deposited and rolled specimens were tensile tested according to ASTM E8 on a Zwick Roell Z100 (Zwick Roell, Ulm, Germany) at a test temperature of 20 °C. Mechanical impact toughness tests were performed according to ASTM E23 on a Zwick Roell RKP450 (Zwick Roell, Ulm, Germany) at −40 °C test temperature. Vickers microhardness was determined at 100 g and 300 g with a FM-310 (Future Tech, Kawasaki, Japan), using the automated software “Thixomet Pro” version 2 (Thixomet, St. Petersburg, Russia).

## 3. Results and Discussion

**Figure 2:**
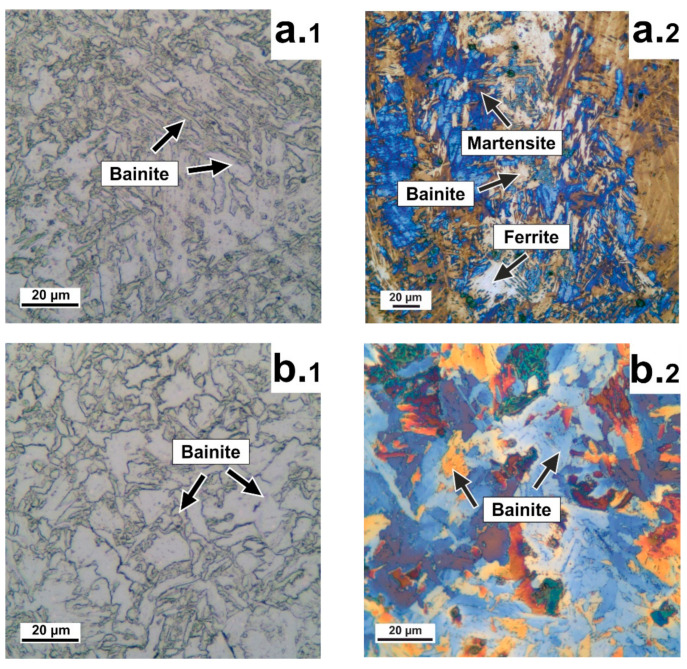

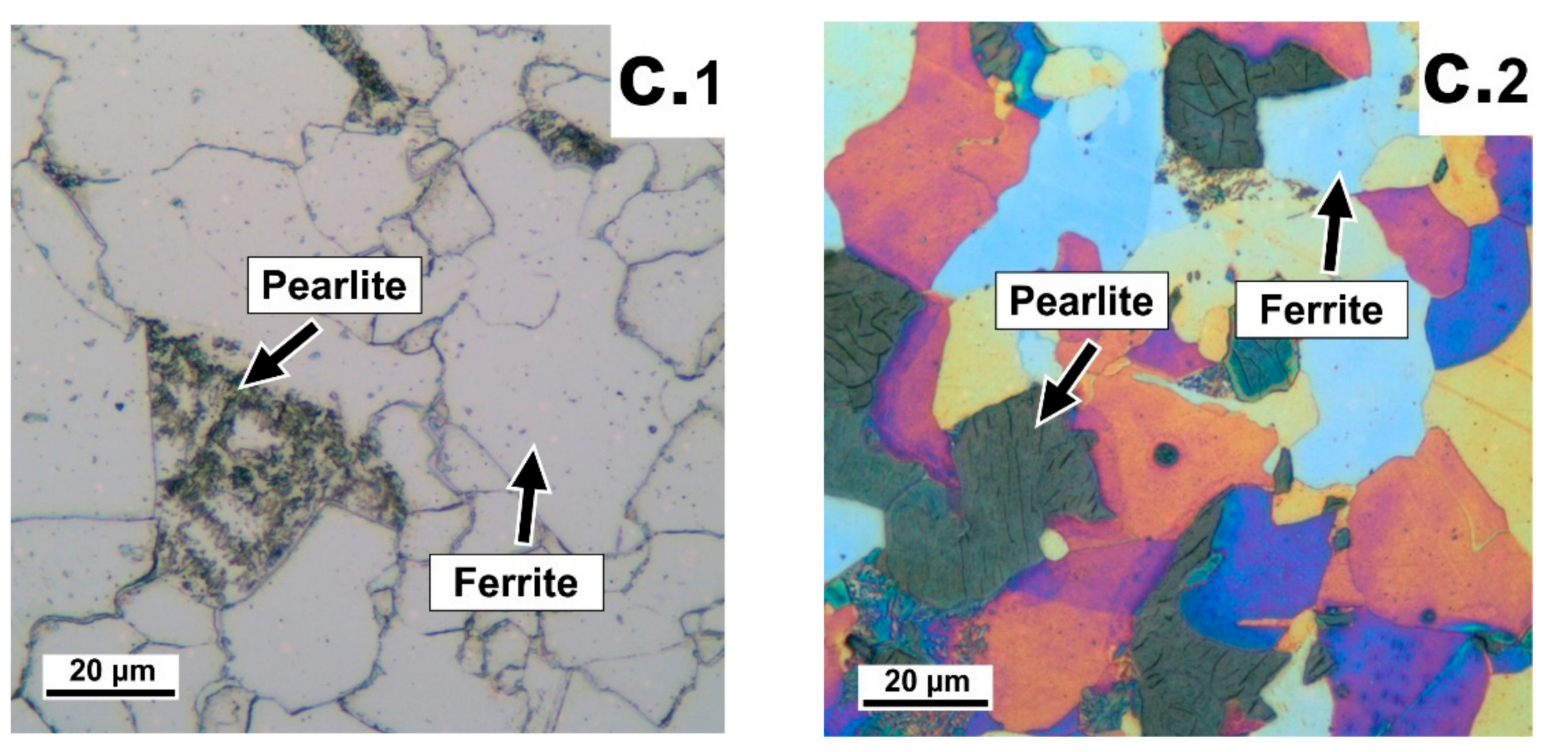
Microstructural observations using an optical microscope: (**a.1**–**c.1**) Nital etching; (**a.2**–**c.2**) Klemm etching; (**a**) Initial state after DLD; (**b**) Mode 1 (DLD +HT); (**c**) Mode 2 (DLD + HT).

Figure 2 shows the microstructural observations using an optical microscope during etching in Nital and Klemm reagent [16], after DLD, Mode 1 (DLD + HT), and Mode 2 (DLD + HT).

When analyzing the microstructure by nital etching (Figure 2(a.1–c.1)), observations do not always provide a clear understanding of the microstructural features as compared with Klemm etching (Figure 2(a.2–c.2)). When analyzing the microstructure in the initial state, i.e., DLD, Figure 2a shows bainitic ferrite of packet type with a microstructure of parallel laths or plates, ferrite, and a small portion of martensite. After HT according to Mode 1 (Figure 2b) the microstructure consisted of irregularly shaped granular bainite grains. After HT according to Mode 2 (Figure 2c), the microstructure after homogenization consisted of ferrite and pearlite.

**Figure 3:**
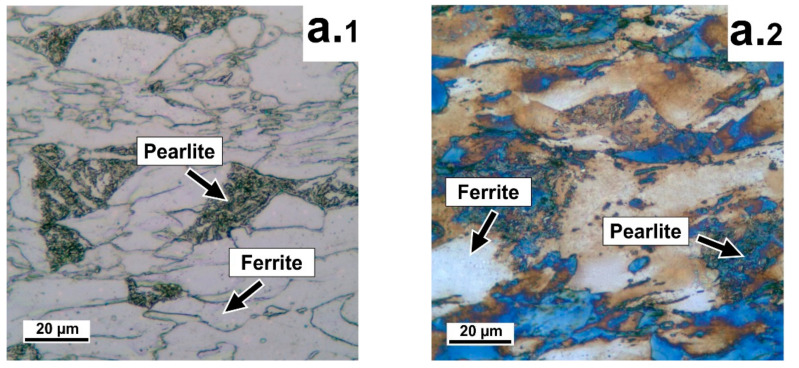

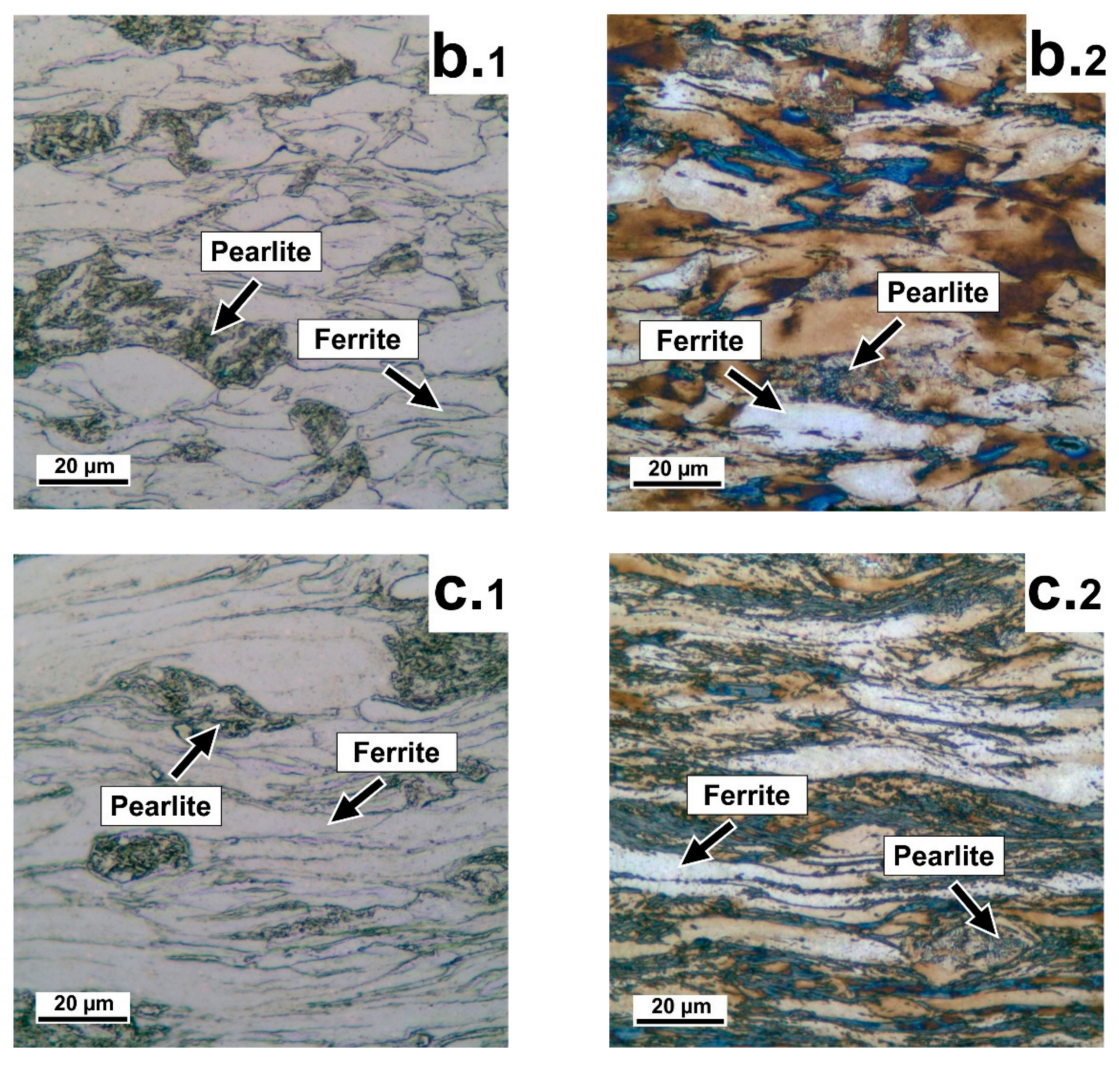
Microstructural observations using an optical microscope, at different degrees of deformation compression: (**a.1**–**c.1**) Nital etching; (**a.2**–**c.2**) Klemm etching; (**a**) 50%; (**b**) 60%; (**c**) 70%.

Figure 3 shows the microstructural observations after cold rolling with different degrees of deformation compression.

The etching of nital (Figure 3(a.1–c.1)) and Klemm reagent (Figure 3(a.2–c.2)) rolled samples gives an identical picture of the structure after cold rolling, consisting of ferrite and pearlite. The phases are present in the material structure in the form of elongated grains of nonequilibrium ferrite and areas of pearlite. With increasing degree of deformation compression, the ferrite structure undergoes more deformation; the microstructure in all samples is nonequilibrium ferrite and pearlite.

Table 2 shows the microhardness results of the individual phases in the structures and the average grain sizes. Structure A is ferrite, structure B is bainite or pearlite.

**Figure 4:**
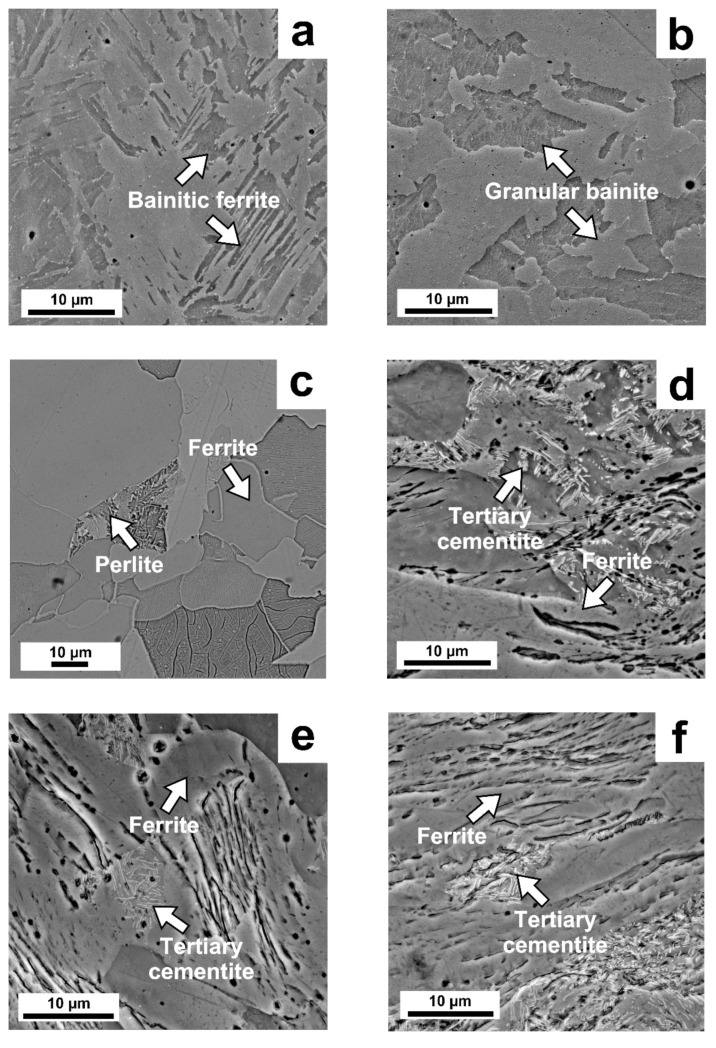
Microstructure of steel 09CrNi2MoCu in various states: (**a**) Initial state after DLD; (**b**) Mode 1 (DLD + HT); (**c**) Mode 2 (DLD + HT); (**d**) 50% degree of deformation compression; (**e**) 60% degree of deformation compression; (**f**) 70% degree of deformation compression.

The microstructure phase composition of steel 09CrNi2MoCu was studied, in detail, with an electron microscope. Figure 4 shows the microstructure of the samples obtained using an electron microscope in various processing conditions.

In the sample initial state after DLD (Figure 4a), the microstructure consisted of bainite ferrite. After HT according to Mode 1, shown in Figure 4b, the microstructure consisted of granular bainite. In the samples after Mode 2 annealing (Figure 4c), the microstructure was a mixture of ferrite and pearlite. Figure 4d–f shows the relief layers in the structure after cold rolling, which are characteristic of a structure after cold rolling. The microstructure of the samples after cold rolling consisted of deformed ferrite, tertiary cementite released from the ferrite phase [16].

Tensile and microhardness tests were performed to evaluate the mechanical properties. The results of these tests are shown in Table 3.

The mechanical properties of deposited specimens in the initial state in the transverse direction are rather low, even after a long HT, the relative elongation increases insignificantly. After cold rolling, the microhardness increased in proportion to the degree of deformation compression. The tensile strength, yield strength, and elongation increased with the degree of reduction. The impact toughness after cold rolling remained at 40–42 J/cm^2^ regardless of the degree of deformation compression. The best result was obtained at 50% deformation compression, where the highest relative elongation values were achieved.

**Figure 5:**
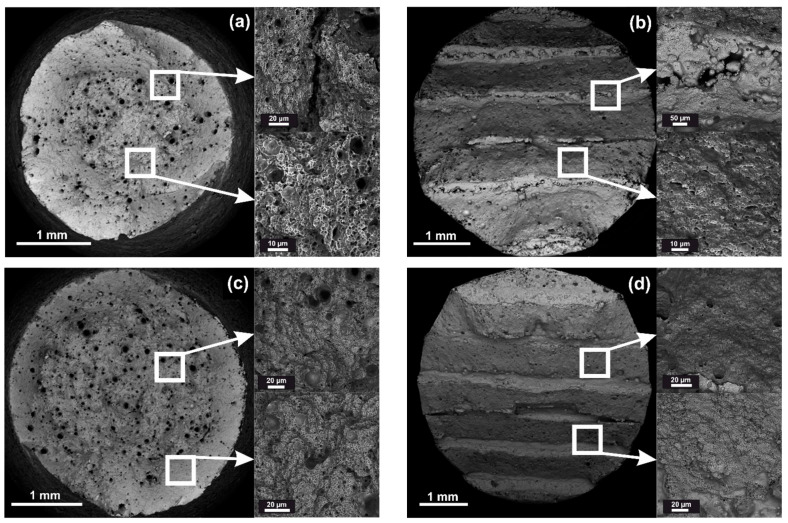
Fracture fractograms after tension of steel 09CrNi2MoCu: (**a**) Initial state after DLD (X orientation); (**b**) initial state after DLD (Z orientation); (**c**) Mode 1, DLD + HT (X orientation); (**d**) Mode 1, DLD + HT (Z orientation).

Figure 5 shows the fractograms of deposited samples after single-axis stretching.

The specimens under study have a pronounced layered character of fracture. During the tests, the metal splits with the formation of depressions up to several millimeters deep, parallel to the deposition plane. The fracture surface of the deposited specimens in the initial state was a viscous fracture with porosity. In the transverse direction, it was a layered ductile fracture with 10% brittle component. After HT in both directions, it was a ductile fracture, with shear pits in the layered section.

**Figure 6:**
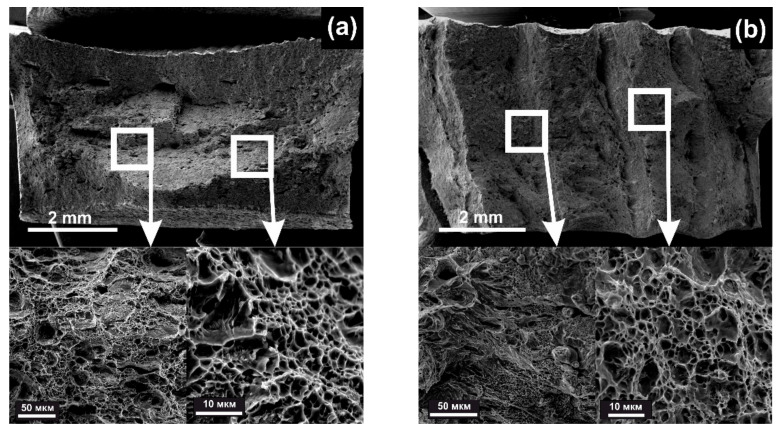

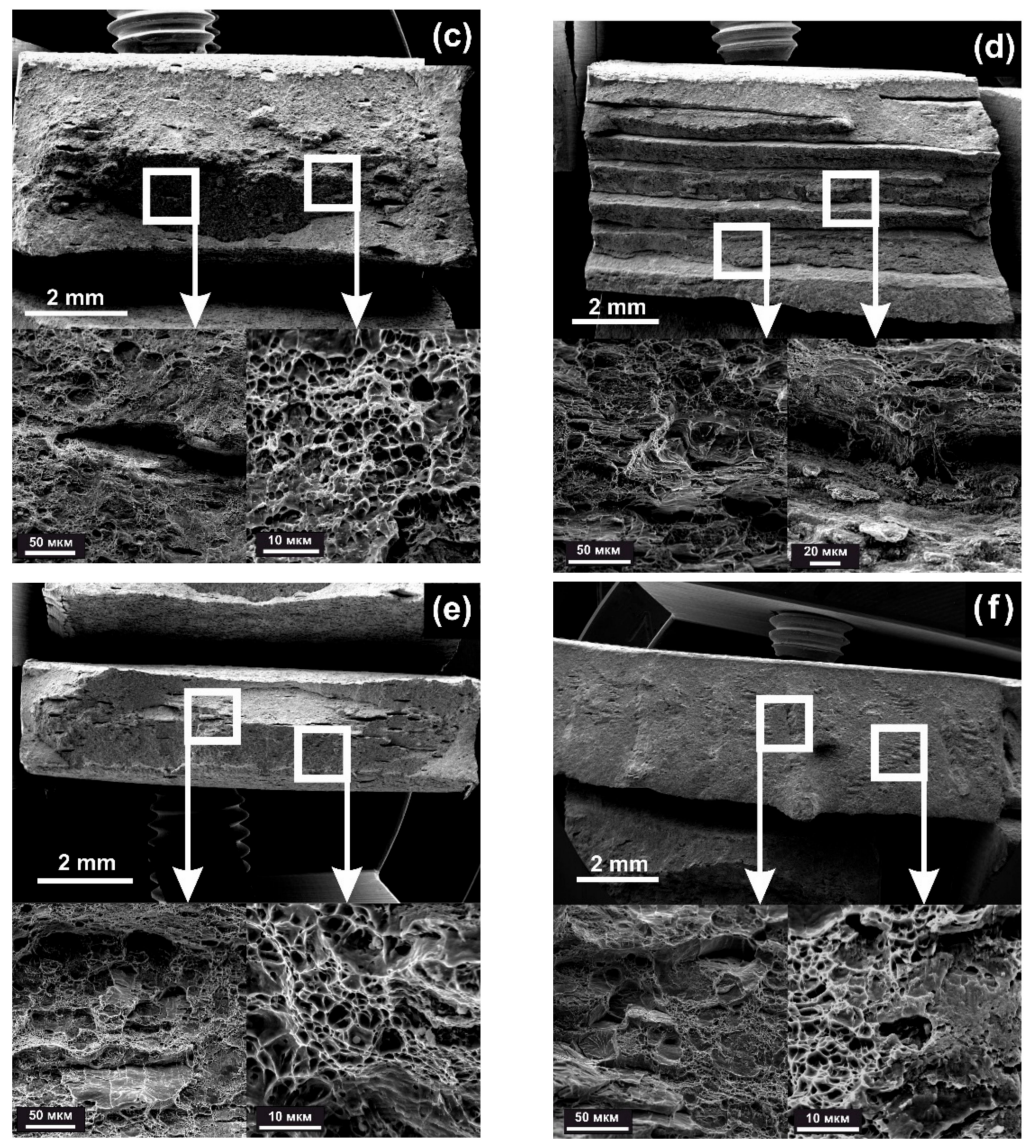
Fractograms of fractures after cold rolling and different degrees of deformation compression: (**a**) 50% (X orientation); (**b**) 50% (Z orientation); (**c**) 60% (X orientation); (**d**) 60% (Z orientation); (**e**) 70% (X orientation); (**f**) 70% (Z orientation).

Figure 6 shows fractograms of fractures after cold plastic deformation.

The fractograms of fractures of rolled samples show elevations and depressions parallel to the rolling plane (Figure 6). The most characteristic relief differences are shown on the samples rolled in the transverse direction. The depth of depressions and the height of elevations is 2–3 mm in the longitudinal (X) samples and 3–5 mm in the transverse (Z) samples.

After cold plastic deformation, pore defects imploded. At 50% strain, the relative elongation increased significantly as compared with the initial deposited state. Further 60% and 70% deformation compression increased the tensile strength and yield strength, but the relative elongation decreased inversely. Increasing the degree of deformation compression had a double effect, i.e., it increased the degree of overlap and elongation of the structure and reduced the density of pore defects.

The fracture surface of the rolled samples in two directions had a different character, depending on the relief. At the upper level of the furrows, a ductile fracture characterized by the presence of dimpled fracture can be observed. In the troughs, ductile fracture with shear pits can be observed, and there is a small fraction of brittle component in the fractures at 60% and 70% of strain, which represents intra-grain spalling.

## 4. Conclusions

In the process of DLD of steel 09CrNi2MoCu, high strength with low ductility and plasticity is achieved. In order to increase the level of mechanical properties, heat treatment has been recommended. However, heat treatment does not always produce uniformly distributed mechanical properties in the longitudinal and transverse directions. In addition, pore-shaped defects are often formed during the deposition of steels by additive methods. Cold rolling can be used to eliminate this defect. In order to obtain a material with high strength and ductility properties, it was necessary to conduct a preliminary heat treatment followed by cold plastic deformation. Impact toughness after cold rolling of the deposited samples was approximately the same regardless of the degree of deformation compression. A significant increase in relative elongation during rolling as compared with the original samples was observed. Minimum values of anisotropy of mechanical properties of deposited samples were achieved at 70% cold rolling, both in the longitudinal and transverse directions.

On the basis of the analysis of the relationships among heat treatment, and the mechanical properties and microstructure of steel 09CrNi2MoCu, the most suitable mode of cold plastic deformation was determined. It was also found that the deposited samples should be rolled in the longitudinal direction. The optimum balanced mechanical properties were achieved at 50% cold rolling deformation compression. At the same time, it was not possible to completely eliminate the anisotropy of the deposited samples; however, after heat treatment and subsequent cold rolling, it was possible to significantly reduce this difference.

## Figures and Tables

**Table 1:** Chemical content of experimental steels, mass % (*).

Steel Grade	Light Element				Cr	Ni	Cu	Mo	Si	Mn	P	Fe
	C	S	O	N								
TU (RU)	0.08–0.11	0.01		≤0.02	0.30–0.70	1.80–2.20	0.4–0.7	0.35	0.17–0.37	0.3–0.6	≤0.015	Bal.
Powder	0.09	0.004	0.026	0.005	0.5	1.93	0.47	0.28	0.25	0.39	≤0.01	

* Researches were performed on the equipment of the Central Complex of scientific equipment «Composition, structure and properties of structural and functional materials» NRC «Kurchatov Institute»—CRISM «Prometey» (financial support of the Ministry of Science and Higher Education—agreement No. 13.CKP.21.0014 (075-11-2021-068). Unique identification number—RF-2296.61321X0014).

**Table 2:** Structural components of the obtained samples.

Type of Treatment	Structure A, (%)	Microhardness, HV^0.1^	Structure B, (%)	Microhardness, HV^0.1^	Average Grain Size, (μm)
Mode 1 (DLD + HT)			100	226–236	3.9
Mode 2 (DLD + HT)	94	166–170	6	223–233	19.5
Cold rolling 50%	92.8	250–260	7.2	351–361	7
Cold rolling 60%	91	256–266	9	371–381	4.9
Cold rolling 70%	90	257–267	10	380–390	4.5

**Table 3:** Mechanical properties of steel 09CrNi2MoCu.

No.	Orientation	Tensile Strength, (Mpa)	Yield Strength, (Mpa)	Relative Elongation, (%)	Impact Toughness, J/cm^2^	Microhardness, HV^0.3^
1. GOST (RU)	N/A	690	512	25.4	98	200–210
2. DLD	X	685	616	21.4	66	251
	Z	637	565.5	7.1	85	
3. DLD + HT	X	665	598	21	116	226
	Z	608	579	15	107	
4. Cold rolling 50%	X	683	460	60	44	257
	Z	700	614	30	41	
5. Cold rolling 60%	X	710	632	51	42	270
	Z	732	656	22	39	
6. Cold rolling 70%	X	785.5	690	28	42	279
	Z	803	711	20	40	

## Data Availability

Not applicable.
